# Basic Optics Underlying Current Intraocular Lenses

**DOI:** 10.3390/jcm14238608

**Published:** 2025-12-04

**Authors:** Yengwoo Son, Seung Pil Bang, Choul Yong Park

**Affiliations:** 1 Department of Ophthalmology, Samsung Medical Center, Sungkyunkwan University School of Medicine, Seoul 06351, Republic of Korea; ysonoph@gmail.com; 2 Department of Ophthalmology, Dongsan Medical Center, School of Medicine, Keimyung University, Daegu 42601, Republic of Korea; gibong87@gmail.com

**Keywords:** multifocal intraocular lens, optics, spherical aberration, chromatic aberration, diffractive lens, extended depth of focus lens

## Abstract

As surgeries using multifocal intraocular lenses (IOLs) to correct both cataracts and presbyopia have become common, it has become essential for clinicians to understand their basic optical characteristics to select the optimal lens for their patients. However, there are relatively few review articles on optics that are directly useful to clinicians who perform surgery on patients. In this paper, we systematically review fundamental concepts, from the basic properties of light, geometric optics, and Gaussian approximation to lens performance metrics like the point spread function and modulation transfer function (MTF), and the clinical implications of spherical and chromatic aberrations. Based on these principles, the mechanisms of major multifocal technologies are explained. We also explore the refractive extended depth of focus lenses, which expand the range of focus by precisely controlling higher-order spherical aberrations. In contrast, diffractive lenses use diffractive kinoforms to split light into multiple foci, and they may also leverage higher diffraction orders to correct chromatic aberration. However, this multifocality involves an optical compromise, often resulting in a reduced overall MTF compared to monofocal IOLs and photic phenomena such as glare and halo. In conclusion, while multifocal IOLs are groundbreaking technology that significantly enhances quality of life by reducing spectacle dependence, this comes at the cost of sacrificing optimal image quality. Therefore, a thorough understanding of these optical principles by ophthalmologists is crucial for selecting the optimal lens according to each patient’s ocular condition and for managing postoperative outcomes.

## 1. Introduction

In recent years, multifocal optical technologies based on various principles have been used in the production of intraocular lenses (IOLs) in the field of ophthalmology [1,2]. These IOLs are referred to as premium IOLs, distinct from standard monofocal IOLs. Multifocal IOLs are categorized based on their principles into diffractive and refractive types, and based on the number of focal points into bifocal, trifocal, or extended depth of focus (EDOF) lenses [1,3,4]. As surgeries using multifocal IOLs to correct both cataracts and presbyopia have gained worldwide popularity, it is imperative for ophthalmic surgeons—including those without formal optics training—to have a basic understanding of the optical characteristics, such as wavefront modulation and focal distribution profiles, of these advanced IOLs. This understanding is paramount for achieving optimal lens selection tailored to individual patient ocular conditions and needs.

This review aims to introduce the fundamental optical principles required to understand the optics of these premium IOLs.

## 2. Understanding Light

**Figure 1:**
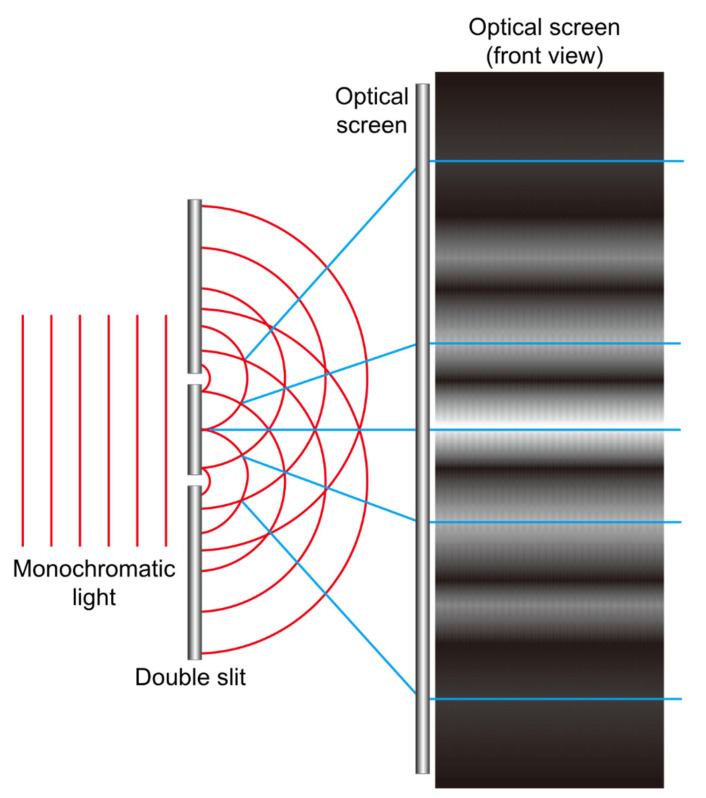
The double slit experiment demonstrates the extreme case of light diffraction, where monochromatic light passing through two adjacent slits produces characteristic interference patterns on a screen via constructive (bright bands) and destructive (dark bands) interference. While this setup fully blocks light between the slits, diffractive intraocular lenses (IOLs) operate differently. In these lenses, the regions between diffractive steps do not completely obstruct light; instead, they act as partial barriers that slow down the propagation of light. As a result, not all light undergoes diffraction—some portions pass straight through the lens and contribute to maintaining distance focus. This dual behavior enables diffractive IOLs to support multiple focal points, including far vision.

Understanding IOL optics requires a fundamental understanding of light. Light is omnipresent in our surroundings. The light we can see is called ‘visible light’, but there are various other electromagnetic waves around us. The dual nature of light—its particle and wave characteristics—has long intrigued the scientific community. Thomas Young’s double-slit experiment provided definitive evidence of the wave nature of light by demonstrating interference patterns exclusively explicable by wave-like propagation. This landmark experiment helped solidify the understanding that light exhibits both particle and wave properties, a foundational concept in modern physics (Figure 1).

Throughout history, various optical theories have been proposed, each attempting to explain aspects of light’s behavior. With the advent of modern science, it is understood that no single optical theory comprehensively describes all phenomena; rather, models such as geometric optics (ray optics), wavefront optics, and diffractive optics are contextually applied to offer a more complete understanding across different scenarios.

**Figure 2:**
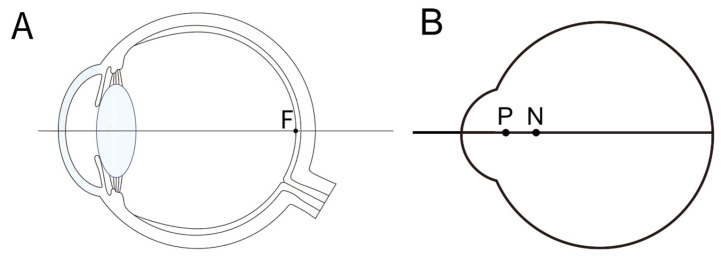
(**A**) Anatomy of an eye designed to explain the optical characteristics of the eye. Light passes through the anterior and posterior surfaces of the cornea, and the anterior and posterior surfaces of the lens, passing through the vitreous cavity, focusing on the fovea (F). (**B**) By combining the cornea and lens into a single refractive surface, we obtain a reduced model eye with the overall refractive index of the optical system being 1.333. The principal point (P) and nodal point (N) are marked.

Our eyes function as complex optical systems, comprising refractive surfaces, including the cornea and lens, alongside transparent media such as aqueous humor and vitreous body, which transmit light to the retina. Figure 2A simplifies the anatomical layout to facilitate understanding of optical phenomena. The eye contains four refractive surfaces: the anterior and posterior corneal surfaces and the anterior and posterior lens surfaces. By combining the refractive powers of the anterior and posterior corneal and lens surfaces, one can model the eye as a single reduced refractive surface (Figure 2B). This simplification excludes intermediate refractions and the aperture stop (formed by the iris as the pupil), which influence light propagation.

Ray optics assumes light travels along straight trajectories, an approximation valid only in homogeneous media. While not perfectly accurate, it effectively approximates actual behavior when the light’s wavelength is much smaller than obstacles or apertures and when the Fresnel number is substantially greater than 1, minimizing diffraction effects. This approximation is useful for many basic optical explanations by allowing image formation to be represented using straight rays. Further mathematical simplifications, known as the Gaussian approximation or paraxial approximation, were introduced by Carl Friedrich Gauss. This approximation facilitates optical system analysis by linearizing ray paths near the optical axis.

## 3. Gaussian Approximation

The Gaussian or paraxial approximation assumes that light rays travel at small angles relative to the optical axis and remain in close proximity to it. Under these conditions, the behavior of rays can be linearized, significantly simplifying the mathematical analysis of their propagation. However, this approximation is only valid for paraxial rays—those near the optical axis—limiting its applicability in systems involving wide angles or large deviations.

Within Gaussian optics, three cardinal points characterize the system geometry: the focal point, the principal point, and the nodal point.

**Figure 3:**
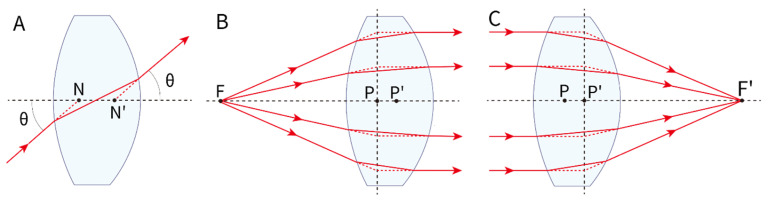
Gaussian geometric optics. (**A**) Rays through the first nodal point (N) emerge through the second nodal point (N′) at the same angle, so rays aimed at a nodal point behave as if not refracted, regardless of lens power. (**B**) In a thick convex lens, light from the focal point emerges parallel; the incident and emergent rays intersect in a plane (front principal plane). The front principal point (P) is where this plane meets the optical axis. (**C**) Conversely, parallel incident rays converge to the focal point after the lens, defining the rear principal plane and rear principal point (P′). F: object position; F’: image position.

First, the nodal point is defined as the point on the optical axis where a ray directed towards it exits the system without angular deviation, i.e., the angles of incidence and refraction are equal for that ray. In thick lenses, two nodal points exist: a front nodal point near the object side and a rear nodal point near the image side. For sufficiently thin lenses, these coalesce into a single nodal point (Figure 3A).

Secondly, the principal point refers to the intersection of the principal plane with the optical axis. For thick lenses, the front principal point is where the extensions of rays refracted from the object focal point and parallel rays intersect in the front principal plane; the rear principal point is similarly defined at the rear principal plane. In thin lenses, the front and rear principal points coincide with the principal point (Figure 3B,C).

**Figure 4:**
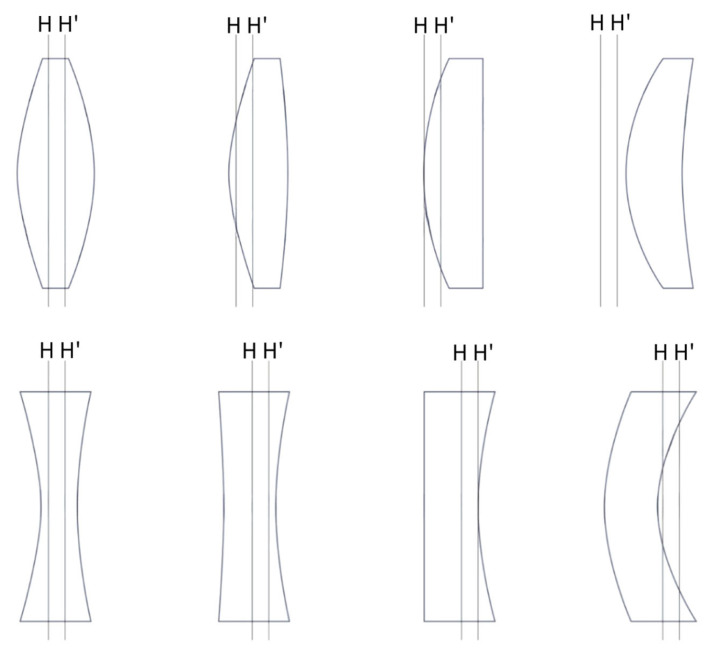
The position of the principal plane varies depending on the shape of the lens. In lenses with various designs of anterior and posterior refractive surfaces, the principal plane generally shifts towards the side with higher refractive power. H: front principal plane; H’: rear principal plane.

Light directed towards the nodal point (N) does not undergo angular deviation, meaning the ray exits the optical system maintaining its angle of incidence. The principal point (P) is defined as the intersection of the principal plane, where the majority of refraction can be approximated to occur in paraxial optics. Figure 4 illustrates how the location of the principal plane depends on the lens geometry and design.

A common misconception is that refraction occurs at the lens center plane; however, the precise location of optical refraction varies based on the lens geometry and thickness [5]. This has important implications for IOL power calculation, where accurate modeling requires considering the effective lens position (ELP)—that is, the refractively effective location of the IOL within the eye postoperatively, which often differs from the anatomical lens position [5,6]. While biometric parameters such as corneal curvature, anterior chamber depth, and axial length can be measured with high precision preoperatively, the uncertainty in ELP remains a principal source of refractive prediction error [7]. The anatomic IOL position (e.g., distance from posterior cornea to anterior IOL surface) does not always correlate precisely with the optically effective position due to IOL shape and design factors (Figure 4 [8,9,10]).

For example, if the principal point of an IOL is significantly anterior to the IOL itself, ELP, which is crucial for refractive accuracy, could be much more anterior than the anterior chamber depth measurement. This tendency is particularly pronounced in high-power lenses [5]. In addition, whether the intraocular lens is designed with front or back vertex powers allows for a more efficient evaluation of its optical behavior within the eye [8]. Among the formulas used for calculating IOL in cataract surgery, the SRK formula has been widely used. Although it has been upgraded to SRK-2 and SRK-T, each IOL brand requires an individual A constant that incorporates design specifics, including haptic angulation, the location of refractive power (anterior or posterior surface or both), and the refractive index of the IOL material. Proper adjustment of the A constant is crucial for accurate postoperative refractive results [3,4].

The principal point is the reference for calculating the refractive power of an optical system, while the nodal point serves as the reference for determining the angle of refraction. In optical instruments, the nodal points are crucial for calculations involving angular magnification. In an idealized system where a convex lens is the sole refractive element and the refractive indices of the media on both sides are identical—such as air—the nodal points coincide with the principal points.

However, in complex biological optical systems like the human eye, characterized by differing refractive indices—in particular, air anteriorly and the aqueous and vitreous humors posteriorly—the nodal points do not coincide with the principal points [11]. Clinically, the nodal points are employed to define the visual axis, which connects the fixation point with the foveola by passing through both nodal points [12,13]. The nodal points are positioned such that a ray entering the eye directed toward the first nodal point emerges from the second nodal point at the same angle relative to the optical axis, thereby maintaining directional integrity. A ray normal to an optical surface traversing the nodal point does so without angular deviation; such a ray exhibits zero transverse chromatic aberration (TCA), a topic elaborated in the following section [14]. Consequently, the visual axis can be identified as the nodal ray that reaches the foveola without chromatic aberration, and for this reason, it is also referred to as the foveal achromatic axis [15,16]. Anatomically, the nodal point is generally located near where the posterior surface of the crystalline lens intersects the visual axis [13].

## 4. Chromatic Aberration

Light consists of a spectrum of colors, commonly known as the visible spectrum, ranging from red to violet. The ray optics model typically assumes monochromatic light, i.e., light of a single wavelength, for simplification. However, in reality, visible light is a composite of multiple wavelengths, each corresponding to a specific color.

When passing through a refractive medium or system, different wavelengths experience wavelength-dependent refraction due to the variation in the refractive index with wavelength, a phenomenon known as dispersion. Shorter wavelengths (e.g., blue or violet light) are refracted more strongly than longer wavelengths (e.g., red light). This difference causes the decomposition of white light into its constituent colors, producing effects such as the rainbow patterns observed when white light passes through a prism.

In technical characterizations of IOLs, optical measurements are conventionally referenced to the green wavelength of approximately 550 nm, which simplifies instrumentation but does not account for polychromatic vision, i.e., perception of full visible spectrum colors [17].

Chromatic aberration observed in human vision is a composite of two principal components: longitudinal chromatic aberration (LCA) and TCA. LCA arises when different wavelengths focus axially at distinct planes along the optical axis, causing a wavelength-dependent focal shift relative to the retina. In contrast, TCA results from wavelength-dependent lateral displacement on the retinal image plane, manifesting as chromatic image blur or color fringing. TCA is often linked to misalignment or decentration of ocular components, which induces lateral chromatic displacement, but fundamentally, it reflects how different wavelengths project on the retinal surface.

**Figure 5:**
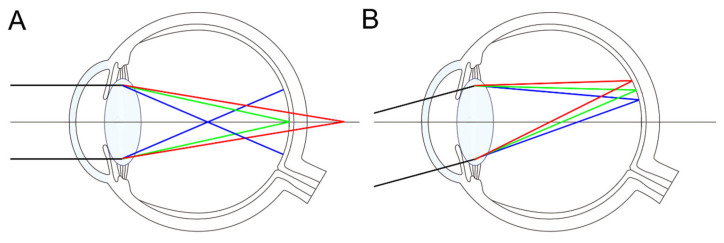
Longitudinal (**A**) and transverse (**B**) chromatic aberration of the eye. (**A**) When green light is focused on the retina, blue light, which has a higher refractive index in ocular media, is focused anterior to the retina, whereas red light, with a lower refractive index, is focused posterior to it. (**B**) Transverse chromatic aberration results in different wavelengths being focused at different lateral positions on the retina.

In clinical ophthalmology, the predominant chromatic aberration of concern is LCA, with the human eye exhibiting approximately 2.5 diopters of LCA across the visible spectrum from red to violet. For instance, if the eye is focused on red light, shorter violet wavelengths focus roughly 2.5 diopters anterior to the retinal plane (Figure 5) [18].

Upon implantation of a 20 diopter IOL targeting emmetropia, the system is optimized for green light, focusing on the macula, while red wavelengths focus posteriorly and blue wavelengths anteriorly to the retina. The clinical red–green test for refractive accuracy derives from this intrinsic ocular LCA. It is known that neural processing in the brain compensates for these optical imperfections, making chromatic aberration less noticeable in everyday vision [19,20,21].

The way to express the chromatic aberration of an optical system is through the Abbe number. The higher the Abbe number, the better the optical system. A lower Abbe number indicates more pronounced chromatic aberration. The Abbe number of the human lens is about 47 [22]. PMMA has a very high Abbe number of 58 [23], and high-end camera lenses have an Abbe number of over 90. Variations in the Abbe number among intraocular lenses (IOLs) can influence visual outcomes due to differences in chromatic aberration. While these optical disparities are theoretically significant, their practical impact on postoperative visual performance is generally minimal and not considered clinically meaningful for the majority of patients. It was known that lenses with a high Abbe number have better modulation transfer function (MTF) than those with a low Abbe number [1,24].

## 5. Optical System Evaluation

A representative method for evaluating the performance of an optical system involves analysis of the point spread function (PSF) and the modulation transfer function (MTF). The PSF describes how an optical system responds to a point source of light, effectively characterizing whether a theoretically infinitesimal point object is imaged as a sharp point or blurred due to system imperfections. Due to the intrinsic wave nature of light, diffraction inevitably occurs in any optical imaging system. This diffraction limits the highest achievable resolution, resulting in what is termed a ‘diffraction-limited image’ [25]. Diffraction typically occurs at the apertures or edges within the optical system, producing characteristic interference patterns that define the system’s impulse response.

**Figure 6:**
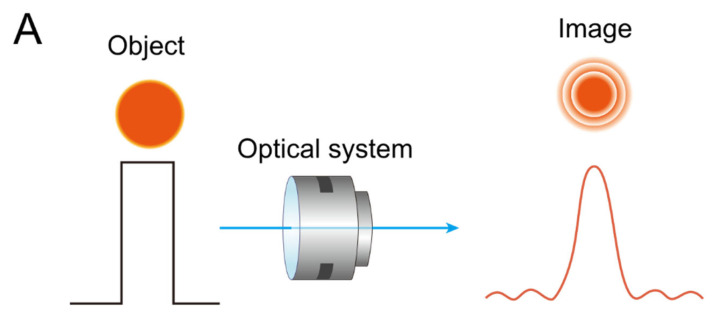

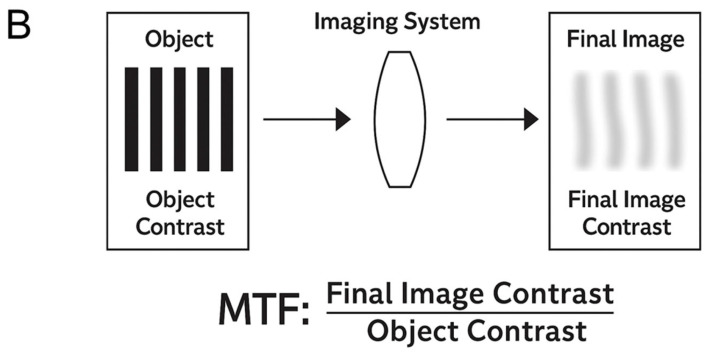
(**A**) As light from a point source (object) (left) passes through an optical system, different images (right) are produced, depending on the characteristics of the point spread function (PSF). Diffraction inherently occurs during this process, particularly when the pupil is small. However, as the pupil size increases, the diffraction effect diminishes, and wavefront aberrations become more prominent. (**B**) Illustration of how spatial frequency content degrades through an optical system, as described by the modulation transfer function (MTF). A square-wave pattern representing high-contrast line pairs (left) passes through a lens, resulting in a blurred sinusoidal output pattern (right).

The PSF can be visualized as the intensity distribution on the image plane from a point source (Figure 6A). An optical system exhibiting a high and sharp central peak intensity at the zero-order diffraction (airy disk) resembles a mountain peak, indicating near-ideal resolution. Conversely, wavefront aberrations broaden the PSF and reduce peak intensity, degrading image quality. Such aberrations can be mathematically modeled using Zernike polynomials [12,26].

The MTF quantitatively evaluates how well an optical system can reproduce contrast from the object to the image, especially for patterns consisting of alternating black and white bars (modulation). Unlike PSF, which describes the impulse response to a point source, the MTF assesses the system’s capability to preserve image contrast at various spatial frequencies. In optical bench simulations for multifocal IOLs, the MTF is commonly analyzed to evaluate the IOL’s performance at different focal distances and spatial frequencies [27,28]. The MTF is fundamental for assessing optical quality and is expressed as the ratio between the ideal modulation (contrast) of an input pattern and the modulation observed in the image formed by the optical system [29]. An MTF value of 1 indicates perfect contrast preservation, representative of an ideal optical system.

Figure 6B illustrates that in a perfect optical system, high-contrast bars on the input side are faithfully replicated on the output side without degradation. However, real optical systems invariably suffer from wavefront aberrations, causing contrast reduction—manifested as poorer blackness of dark regions and whiteness of bright regions in images. This results in a diminished MTF at certain spatial frequencies [12,27].

For eyes implanted with multifocal IOLs, the overall MTF tends to be lower than that of monofocal IOLs, especially at far focus. Clinically, this can translate to slightly blurred or less distinct views of detailed objects such as road signs, distant scenes, or traffic lights. Such contrast and resolution degradation is more pronounced under low-light conditions (e.g., dusk or nighttime), affecting the patient’s quality of vision in these scenarios.

MTF is mathematically the magnitude of the optical transfer function (OTF), which is the Fourier transform of the PSF, and provides a comprehensive characterization of an optical system’s spatial frequency response and image quality [29,30].

It is noteworthy that the MTF of an optical system can be evaluated using either through-focus or through-frequency modes. In through-focus mode, MTF is measured as a function of defocus by systematically varying the image plane [31]. This approach is particularly valuable for translating optical bench data into clinically relevant insights, as defocus corresponds to intermediate and near vision ranges. In contrast, through-frequency mode assesses MTF at a fixed optical power—typically at the far-focus position—across different spatial frequencies, such as 20, 50, and 100 line pairs per millimeter (lp/mm), or their equivalents in cycles per degree [32]. By analyzing lens performance across varying defocus levels and spatial frequencies, clinicians gain a deeper understanding of how different IOL designs perform at multiple viewing distances. This, in turn, supports more informed decision-making when selecting the most suitable lens tailored to each patient’s unique visual needs [33,34,35].

## 6. Spherical Aberration

Spherical aberration is a type of wavefront aberration that arises when rays passing through zones farther from the optical axis of a spherical surface are refracted by different amounts compared to paraxial rays passing near the center. This deviation results in a departure from the ideal focusing capability expected from a perfect optical system. The phenomenon is quantitatively defined as the wavefront deviation, expressed as the optical path difference between the actual wavefront emerging from the optical system and an ideal spherical reference wavefront, measured across the entrance pupil. When marginal rays are refracted more strongly than paraxial rays, causing them to focus closer to the lens than predicted by the ideal model, the system exhibits positive spherical aberration. Conversely, when marginal rays are refracted less strongly and focus farther away, it is referred to as negative spherical aberration.

It is known that the average spherical aberration of the human cornea is about 0.30 µm, while the natural crystalline lens contributes about −0.20 µm of spherical aberration for a 6 mm pupil diameter [36,37]. Therefore, the total spherical aberration of the human eye is about 0.10 µm. However, spherical aberration of the natural crystalline lens changes with aging, going from negative to positive as cataracts develop [38,39]. This positive spherical aberration of the cataractous lens adds to that of the cornea, impairing the visual quality in elderly patients. However, increased spherical aberrations may extend the depth of focus, potentially enhancing intermediate vision at the expense of some distance visual quality [40,41].

In the past, spherical IOLs were the only options for cataract surgery. However, aspheric IOLs were introduced to compensate for the positive spherical aberration of the cornea with various designs such as an anterior prolate surface (Tecnis, Johnson & Johnson, New Brunwick, NJ, USA), a posterior prolate surface (Acrysof IQ, Alcon, Fort Worth, TX, USA), or with both anterior and posterior prolate surfaces (Akreos AO, SofPort AO and L161 AO, Bausch & Lomb, Vaughan, ON, Canada) [42].

Aspheric IOLs can be divided into two categories. One with negative spherical aberration mimicking natural crystalline lens (AcrySof IQ SN60WF, Tecnis ZCB00, and Vivinex or models 250 and 251, Hoya, Tokyo, Japan) and the other with no spherical aberration, i.e., aberration-free aspheric design (Akreos AO, Bausch + Lomb; enVista MX60, Bausch + Lomb; or AT LARA 929M/MP, Zeiss, Oberkochen, Germany) [42]. IOLs with negative spherical aberration have the advantage of improving patients’ vision by fully compensating for the positive spherical aberration of the cornea. However, they are more sensitive to lens tilt or decentration, which can increase the risk of postoperative visual degradation compared to aberration-free aspheric IOL [43,44].

Some IOLs utilize negative spherical aberration to enhance intermediate and near vision. The principle is that during near work, when the pupil constricts, IOLs with negative spherical aberration use the lens’s central part with relatively higher refractive power, making it easier to distinguish objects. By mathematical approximation, 0.25 µm of negative spherical aberration with a 6 mm pupil diameter can result in at least 0.75 diopters of myopic defocus, and 0.4 µm of negative spherical aberration can induce 2.0 diopters of myopic defocus, thereby enhancing reading ability [45]. Eyhance ICB00 (Johnson & Johnson) is one of the first enhanced monofocal IOLs incorporating negative spherical aberration, which means an IOL designed to improve intermediate vision while maintaining similar distance vision as a monofocal IOL [46].

Building on this principle, newer non-diffractive EDOF IOLs, such as the PureSee, utilize a more sophisticated method of modulating higher-order aberrations to extend the depth of focus. A core principle of these IOLs is the combination of negative 4th-order spherical aberration and positive 6th-order spherical aberration of opposite signs [47,48]. This combination of opposite signs has been shown to increase the depth of focus more effectively than using a single aberration or combining them with the same sign. Applying this principle, the PureSee IOL features a subtle power change on its central optic, which creates an annular zone with approximately 3 diopters of additional power around the central area [49]. Schmid et al. evaluated the optical properties of PureSee IOL using an optic bench. They found the IOL has negative spherical aberration of −0.66 µm and a steeply sloping annular myopic zone with 3 diopter power at the radial position about 1 mm from the IOL center. The calculated visual acuity using MTF was over 0.8 at 50 cm and over 0.63 at 40 cm [49].

However, this design is not always advantageous. While the refractive profile performs well when the IOL is properly centered, it may increase patient discomfort in cases of IOL decentration or with an eccentric pupil [50,51], due to the induction of coma-like aberrations. A novel refractive design incorporating a radially periodic power profile has been proposed as a potential solution to this limitation [52]. Moreover, long-term exposure to an individual’s habitual spherical aberration may lead to neural adaptation that modulates the perception of defocus, thereby influencing subjective depth of focus [53]. Notably, the aging visual system has demonstrated the capacity for re-adaptation following changes in ocular optics, such as after cataract surgery, with progressive improvements in visual performance observed in pseudophakic eyes over time [54]. These neural compensation mechanisms may play a critical role in determining the clinical effectiveness of presbyopia-correcting IOLs and other optical interventions.

## 7. Diffractive Optics for IOLs

### 7.1. Diffractive Phenomenon

**Figure 7:**
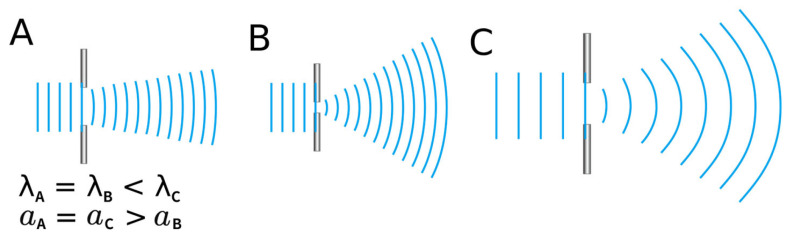
The amount of diffraction a wave experiences is determined by the size of the gap it encounters and the wavelength of the wave itself. In the figure, light travels from left to right. A narrower gap (as in (**A**) compared to (**B**)) results in greater diffraction. Similarly, for waves passing through the same gap, a longer wavelength (as in (**A**) compared to (**C**)) leads to more pronounced diffraction. Therefore, red light, which has a longer wavelength than blue light, undergoes a greater degree of diffraction. *λ*: wavelength; *a*: slit width.

Currently, the most popular multifocal IOLs are diffractive IOLs. As we discussed, light possesses both particle and wave properties, allowing it to diffract like waves. As shown in Figure 7, the diffraction phenomenon of waves depends on the wavelength of the wave and the size of the obstacle. For a given wavelength, the smaller the gap, and for a given gap, the longer the wavelength, the more the path is bent due to diffraction. The resulting focal length due to diffraction can be simply expressed mathematically, where r is the radius of the gap and *λ* (lambda) is the wavelength. This relationship can be described by the equation f = r^2^/*λ*, where f represents the effective focal length generated by diffraction. Essentially, the longer the wavelength and the smaller the gap, the shorter the focal length, meaning the diopter, a unit of power, increases.

Conceptually, diffraction can be understood in multiple ways. When light encounters a discontinuity or edge, it slows down and changes direction—this is the essence of diffraction. Alternatively, adopting a particle-based view of light (photons) can provide a more intuitive understanding: photons comprising a light wavefront may be redirected into different directions upon interacting with diffractive steps. A useful analogy is the redirection of air molecules by dashboard air vents in a car—adjusting the louvers redirects air flow, much like diffractive elements redirect photons into separate foci. An excellent review written from the perspective of optical researchers was previously published [2]. It is highly specialized and mathematical, and is recommended for those seeking a deeper understanding of diffractive optics [2].

### 7.2. Fresnel Lenses

**Figure 8:**
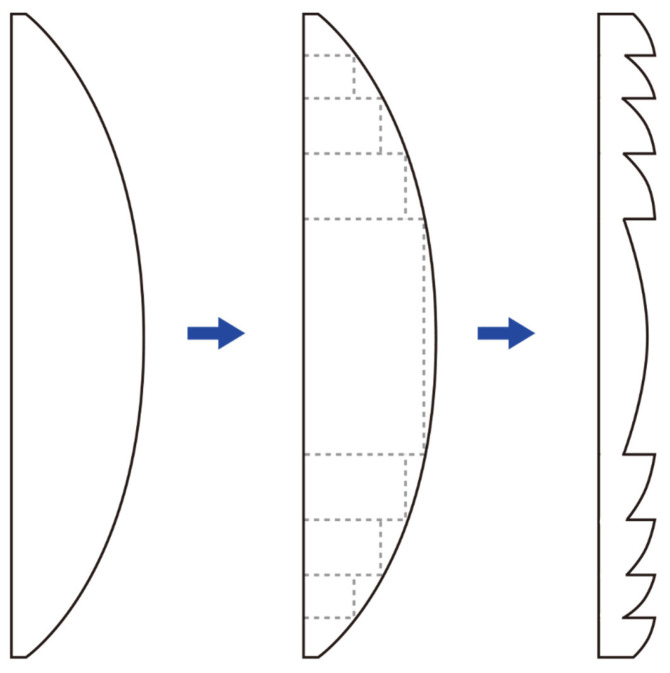
In a conventional convex lens (**left**), removing the sections that do not contribute to refraction (dashed rectangular areas) results in a Fresnel lens (**right**) with the same refractive power but reduced thickness. This design allows the Fresnel lens to maintain the functionality of a traditional lens while being thinner and lighter.

To better understand diffractive optical elements, it is instructive to begin with the principle of Fresnel lenses —a technology developed by the French physicist Augustin-Jean Fresnel (1788–1827). The basic principle is illustrated in Figure 8.

A conventional convex lens can be conceptually partitioned into two components: the curved refractive surface that directs light rays, and the non-refractive bulk material that primarily contributes to the lens’s thickness without affecting its optical power. By excising these non-refractive portions while preserving the refractive segments, a thin Fresnel lens is constructed, significantly reducing material volume and weight without compromising optical power. There are no strict limitations on where the zone boundaries must be placed in a Fresnel lens, allowing flexibility depending on the application. One common design approach is to maintain constant step heights across zones. In this case, the zone widths must vary to preserve the desired optical path difference. Alternatively, the zones can be designed with constant widths, which then requires the step heights to vary accordingly [55]. The same principle can also be applied to make Fresnel prism lenses. In modern ophthalmology, temporary Fresnel prisms or lenses are sometimes affixed to spectacle lenses, typically employing zone widths of at least 1.0 mm to minimize diffractive side effects.

The concentric ring structures of Fresnel lenses closely resemble those observed in diffractive multifocal intraocular lenses (IOLs). Optical elements generally exhibit both refractive and diffractive properties, with their dominant behavior depending on the relative size of the structural features to the wavelength of incident light. When features exceed the wavelength significantly, refractive effects prevail; when feature sizes approach the wavelength, diffractive effects dominate. In Fresnel lenses, as the grating or step height decreases, diffractive behavior becomes increasingly pronounced, and the lens transitions from acting primarily as a refractive element to predominantly exhibiting diffractive behavior.

Practically, manufacturing a true diffractive lens with extremely small-scale curved grating features is challenging due to technical limitations. To facilitate production, these gratings are often fabricated with a blazed (slanted) profile, creating what is known as an approximate Fresnel lens. This blaze design optimizes diffraction efficiency by directing light predominantly into a desired diffraction order. At sufficiently small feature sizes, the optical surface no longer behaves conventionally as refractive but induces controlled wavefront modulation through diffraction, critical for multifocal IOL performance.

### 7.3. Distribution of Light in Diffraction

**Figure 9:**
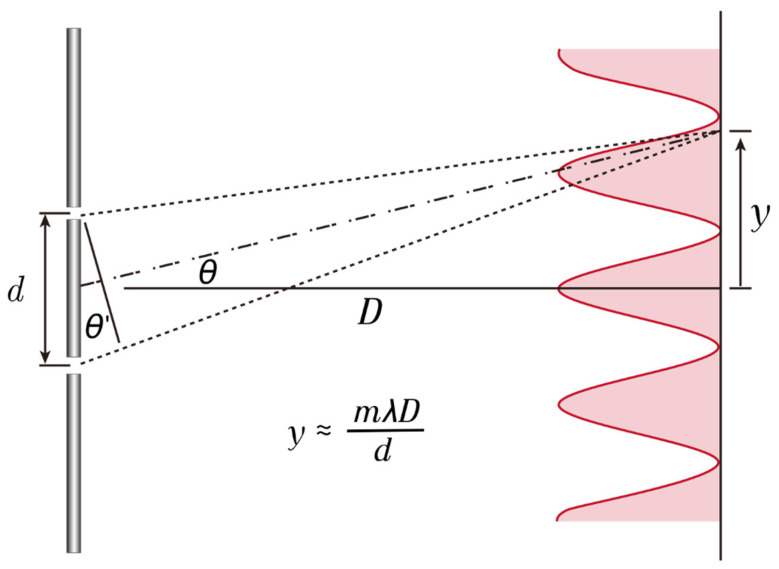
This diagram illustrates the location of the diffractive focus. If we assume that the distance to the screen (D) is much greater than the distance between the double slits (d); then, the light passing through the double slits undergoes diffraction due to interference, causing the distance of m order of maxima (y) to be inversely proportional to the slit spacing (d) and directly proportional to the wavelength of the light (*λ*).

The distribution of light on a screen after passing through two slits is shown in Figure 9. The optical axis corresponds to the 0th order, and as you move away from the optical axis, bright and dark patterns alternate, designated as the 1st order, 2nd order, and so on. There is an optical formula that determines the positions where the peak intensities of the 0th, 1st, and 2nd orders will appear based on the wavelength of light and the distance between the slits and the screen. If the slit spacing (d) is narrowed, the deviation of the light path (y) increases, making the 1st order diffraction pattern appear to refract at a greater angle than the direction of light travel. Conversely, if the slit spacing is widened, the numerator in the formula increases, causing the 1st order diffraction pattern to appear to refract at a smaller angle.

In a pure kinoform lens, the 0th order light remains undiffracted and travels straight without forming a focus; however, in real multifocal IOLs, both diffraction and refraction occur simultaneously, causing the 0th order light to primarily serve the far focus. In diffractive multifocal lenses, the slit spacing (d) corresponds to the spacing of the kinoform, and reducing this spacing increases the additional optical power by deviating light at a relatively larger angle. In other words, the distribution of light in the 1st order indicates a shorter focal length from the optical axis, with the 2nd order having an even shorter focal length [56,57].

**Figure 10:**
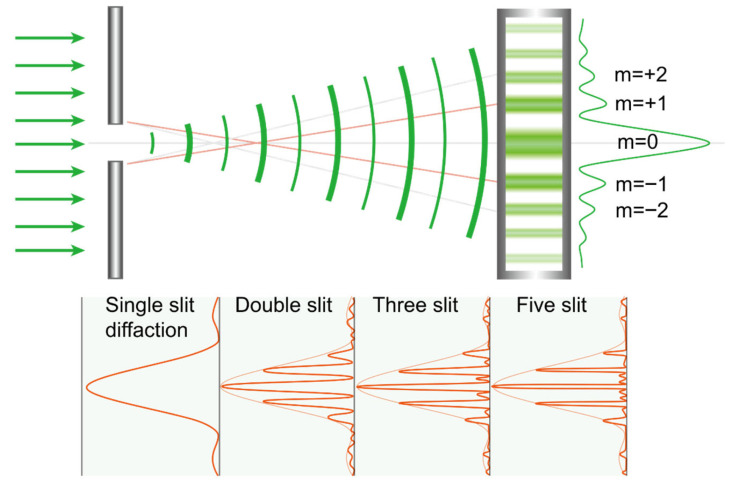
As increasing the number of slits makes the diffracted light’s energy peaks at the intended positions more pronounced, in a multifocal intraocular lens, enlarging the pupil increases the number of concentric rings through which light passes, producing a similar effect. m: order of maxima.

Moreover, compared to a single slit, an increase in the number of diffractive gratings results in a narrower and more concentrated energy distribution at each maximum. Therefore, the greater the number of concentric rings in a diffractive IOL, the more distinct the energy peak at the intended focal point. This characteristic explains why energy distribution may vary depending on pupil size after inserting a diffractive IOL [58] (Figure 10).

### 7.4. Diffractive Optic Element

The structures that primarily cause light diffraction are called diffractive optic elements, also known as gratings or kinoform. When the grating has a pointed shape, it is referred to as a kinoform. A kinoform is a phase-only diffractive optical element that implements a continuous phase profile designed to steer nearly all incident energy into a desired diffraction wavefront or focal pattern, whereas a grating is a generally periodic structure that distributes light into multiple diffraction orders according to the grating equation. Diffractive IOLs like Panoptix (Alcon), Synergy (Johnson & Johnson), and Odyssey (Johnson & Johnson) have concentric rings of various sizes, which act as kinoforms.

**Figure 11:**
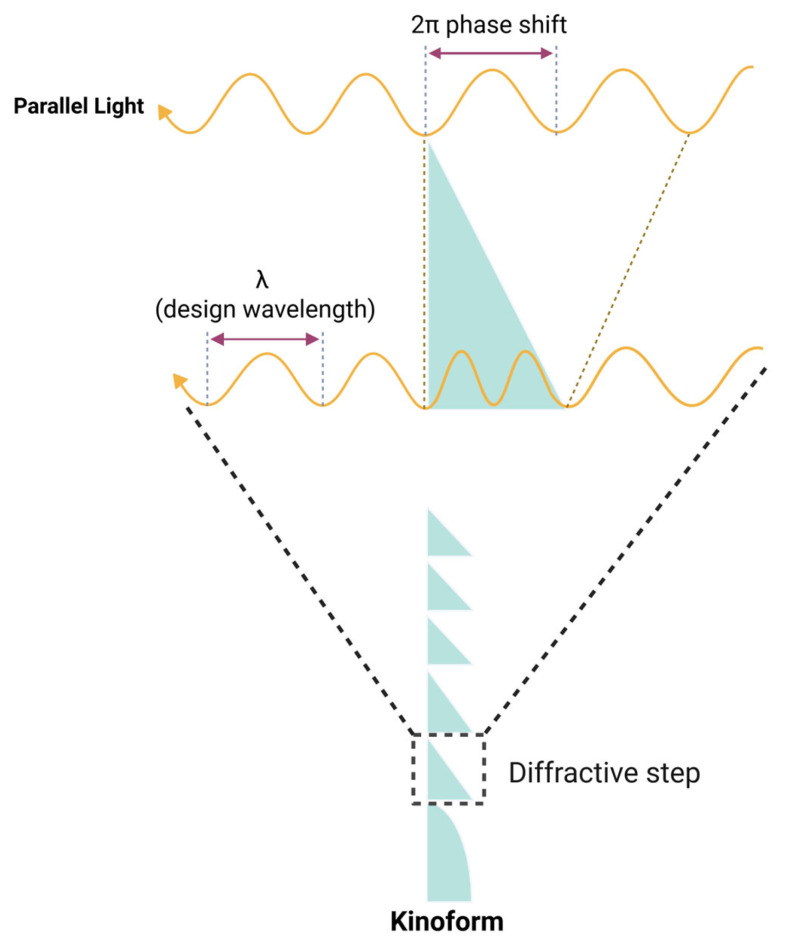
As light passes through a kinoform, a phase shift is induced due to the variation in optical path length between the base and apex of the structure. The diagram illustrates a case where the phase difference between light passing through the base and the apex equals one full wavelength (2π). Unlike square gratings, the kinoform’s unique geometry becomes significant here: the base induces the maximum phase shift, while the phase shift gradually diminishes toward the apex. This creates a continuous phase-shifting gradient within the kinoform, allowing it to focus light efficiently toward desired focal points.

The explanation of light path changes occurring in diffractive optics is thoroughly detailed in the previous studies [56,59]. In diffractive optics, the light path alteration results from a phase delay introduced by microstructured features on the optical element. As depicted in Figure 11, light passing through the kinoform structure of an IOL encounters a phase delay primarily in the thicker base regions, due to the higher refractive index of the IOL material relative to the aqueous humor. This phase delay causes the light to traverse the thicker base to propagate more slowly, creating a relative phase lag compared to light passing through the kinoform’s thinner apex areas. By mapping the wavefronts before and after transmission, it is evident that the wavefront bends toward the kinoform base, signifying a precise phase modulation mechanism that controls light direction.

This refractive index contrast, combined with the kinoform’s geometry, forms the basis for the diffractive mechanism that enables multifocality in IOLs, where the phase delay distribution manipulates the wavefront to direct light to multiple focal points with high precision [2,60].

### 7.5. From Fresnel Lenses to Monofocal Diffractive Lenses

**Figure 12:**
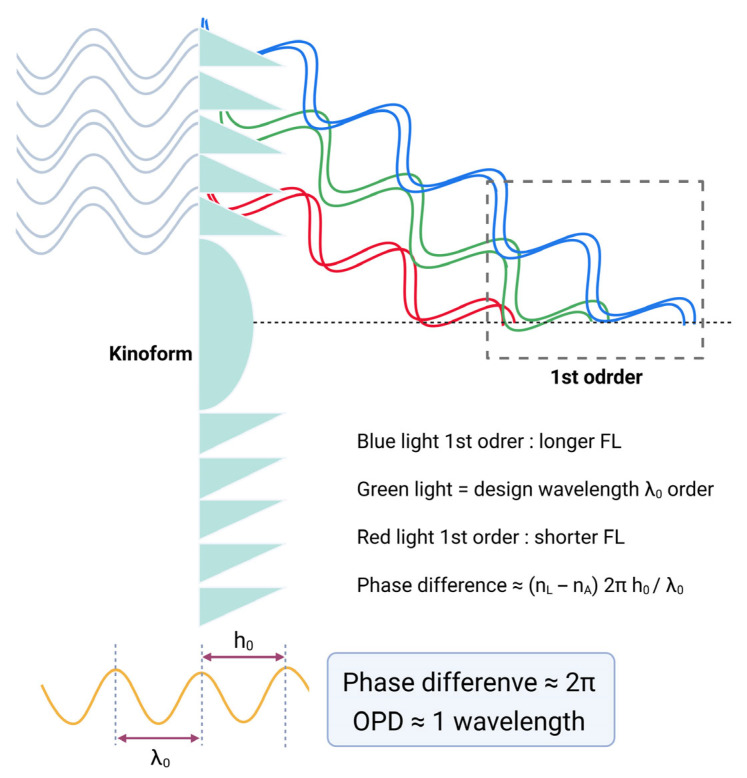
Diffraction behavior of a monofocal diffractive lens as a function of phase difference. When the phase difference equals 2π (OPD ≈ 1 *λ*), the zero-order beam is suppressed, and only first-order diffraction occurs. As discussed later, blue light has a longer focal length (FL), while red light has a shorter focal length (FL). Phase difference can be calculated using an equation: phase difference ≈ (n_L_ − n_A_) 2πh_0_/*λ*_0_, n_L_: refractive index of lens, n_A_: refractive index of aqueous, h_0_: height of kinoform, *λ*_0_: wavelength of light. OPD = optical pathway difference.

A monofocal diffractive lens can be regarded as a limiting case of the Fresnel lens. In a first-order diffractive lens, the zone boundaries must align with the Fresnel zones corresponding to full-wavelength optical path differences, and the phase delay at each step must equal one full wavelength (Figure 12). To achieve the design of a monofocal diffractive lens, the radius r of the boundary for the *i*th diffractive zone is given by the equation:

$${r_{i}}^{2}=2i\cdot \lambda_{0}\cdot f$$

where *λ*_0_ is the design wavelength, and *f* is the focal length of the lens. This phase-controlled structure, sometimes referred to as a kinoform, is not typical of refractive Fresnel lenses and has become more practical with the advent of high-precision lathes, allowing for meticulous control of phase and zone placement.

Although Fresnel lenses and monofocal diffractive lenses appear similar at a glance, they function quite differently. In a refractive Fresnel lens, each zone refracts light independently, often with arbitrary phase discontinuities between adjacent zones, leading to unintended diffraction artifacts. In contrast, a diffractive lens functions as a coherent system, with all zones contributing collectively to focus light. Its zone boundaries are precisely placed where the optical path increases by one wavelength, and phase shifts are carefully engineered, enabling high-quality image formation. The tight control over Fresnel zones and precise phase modulation—down to sub-wavelength accuracy—underpins the performance of modern diffractive IOLs.

### 7.6. From Monofocal to Multifocal Diffractive Lenses

**Figure 13:**
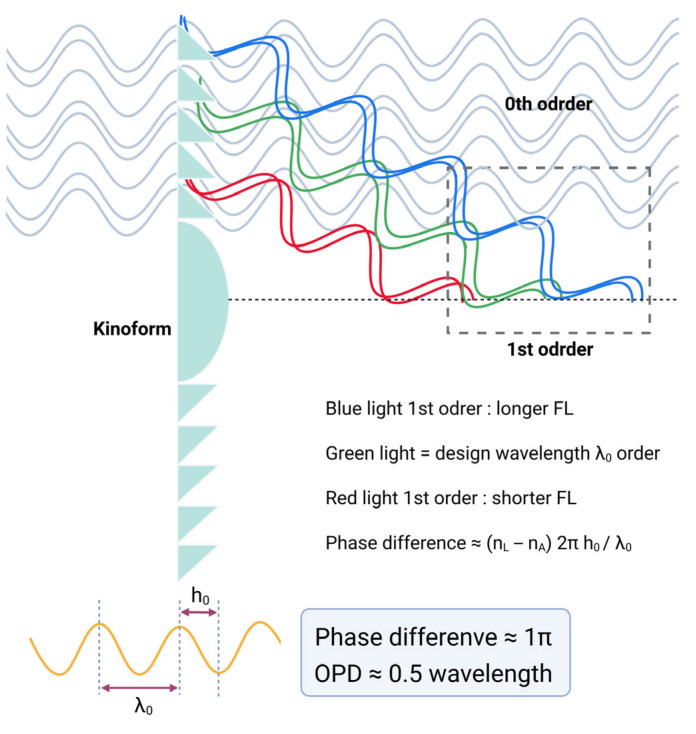
Diffraction behavior of a bifocal diffractive lens as a function of phase difference. When the phase difference equals half a wavelength (OPD ≈ 0.5 *λ*), equal fractions of light are directed into the zero- and first-order beams. OPD = optical pathway difference.

The primary distinction between a monofocal diffractive lens and a bifocal diffractive lens lies in the phase delay at the kinoform. For monofocal lenses, the phase delay is usually one wavelength, whereas for bifocal lenses, it is typically half a wavelength (Figure 13). The kinoform zones do not operate through classical refraction; thus, the behavior of multifocal diffractive lenses cannot be accurately modeled by conventional refractive optics. If traced as refracted rays, the light would incorrectly appear to focus at an intermediate point between the two primary focal points. However, in reality, the diffractive structure splits the incident light, precisely directing it to the respective foci, with negligible energy at the midpoint, enabling simultaneous clear vision at multiple distances.

The commonly adopted kinoform height of most bifocal diffractive IOLs we use is typically 2 µm [34,60,61]. Most of the IOLs we use have a refractive index of around 1.50, and the aqueous humor has a refractive index of 1.336. Since the basic wavelength of light considered in optical evaluation of IOL is the green wavelength of 550 nm, if the kinoform height is about 2 µm, considering the refractive index of the IOL material, the light is evenly distributed between the 0th order and the 1st order with π phase difference using the phase difference equation (phase difference ≈ (n_L_ − n_A_) 2πh_0_/*λ*_0_, n_L_: refractive index of lens, n_A_: refractive index of aqueous, h_0_: height of kinoform, *λ*_0_: wavelength of light) as in Figure 13.

At the design wavelength (550 nm), 40.5% of the incoming light energy is directed to each of the two primary focal points, while around 19% is lost to higher diffractive orders [34,62]. For instance, with an add power of 3.0 diopters, higher-order foci (e.g., ±6 D, ±9 D) represent locations where the optical path differences increase by multiples of the wavelength (2*λ*, 3*λ*, etc.). These are inevitable byproducts of light’s interaction with the diffractive profile. Therefore, a total energy transmission of 81%—with 40.5% to each focus—is the maximum theoretical efficiency achievable in a full-aperture bifocal diffractive system. It is not physically possible to direct 50% of the light to each of two foci using diffractive optics.

**Figure 14:**
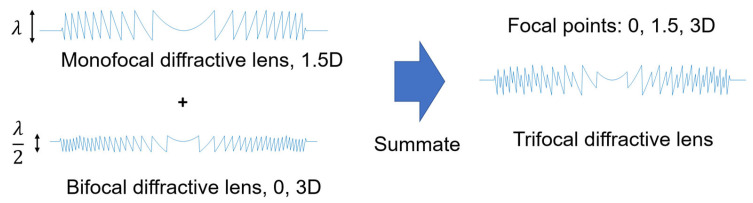
Design concept of a trifocal diffractive lens. By combining two diffractive optics with different kinoform heights, it is possible to fabricate various types of diffractive multifocal intraocular lenses (IOLs) that provide dual, trifocal, or even quadrifocal functionality.

The development of trifocal IOLs has significantly advanced cataract surgery by allowing patients to achieve functional vision at near, intermediate, and far distances—often without the need for additional corrective eyewear. Trifocal IOLs achieve this by incorporating two distinct diffractive profiles that distribute light efficiently across the three focal points. In essence, the optical design involves first constructing a monofocal diffractive profile and a bifocal diffractive profile, each with different phase delays. These two profiles are then superimposed onto the lens surface to create trifocal functionality (Figure 14).

### 7.7. Higher Order Diffractive Lenses: Chromatic Aberration Compensation

Typical diffractive bifocal IOLs, featuring kinoform step heights of approximately 2 µm, operate by exploiting the 0th and the 1st diffraction orders to generate distinct focal points for distance and near vision, respectively. In such designs, the 0th diffraction order predominantly facilitates distance vision by utilizing the high optical power of the refractive base lens to project collimated light from far objects onto the retinal plane. Conversely, the +1st diffraction order is harnessed for near vision, combining the refractive power of the carrier lens with the phase shift introduced by the diffractive profile to create an additional focal point corresponding to near accommodation.

**Figure 15:**
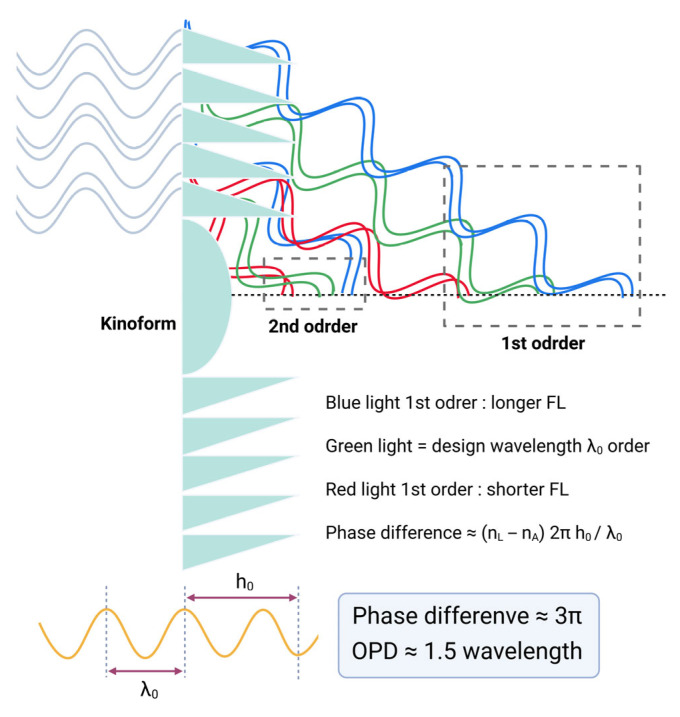
Diffraction behavior of a bifocal diffractive lens as a function of larger phase difference. When the phase difference equals 3π (OPD ≈ 1.5 *λ*), equal fractions of light are directed into the 1st-order and 2nd-order beams. OPD = optical pathway difference.

If the kinoform step height is increased to approximately 6 µm, introducing a phase delay close to one and a half wavelengths (3π), light energy is predominantly diffracted into the 1st and 2nd diffraction orders [57,63]. This larger step height modifies the diffraction efficiency distribution, shifting more optical power into the 1st and 2nd orders, which correspond to secondary and tertiary focal points in multifocal IOL designs. Such a configuration can be employed in trifocal IOLs where the 1st order typically serves near vision, and the 2nd order provides intermediate focus, optimizing the visual performance across multiple focal distances (Figure 15) [64,65,66].

As previously discussed, in refractive optics, longer wavelengths bend less when passing through a medium—an effect visually demonstrated by the dispersion of light in a prism. In contrast, diffractive optics exhibit opposite behavior. Due to the periodic nature of diffractive structures, their focusing power increases linearly with wavelength—meaning it is higher for longer wavelengths and lower for shorter ones. As a result, positive diffraction orders introduce LCA that is opposite in sign to the LCA produced by the cornea and the refractive carrier lens. This property can be advantageous in IOL design, as it offers a means to partially compensate for the eye’s natural chromatic aberration, and this approach is commonly referred to as chromatic aberration-correcting technology [67,68]. The LCA induced by a diffractive structure is described by the following equation:

$$LCA=\frac{-m\cdot \left|\Delta \lambda \right|}{\lambda_{0}}\cdot P\left(\lambda_{0}\right)$$

where *m* represents the diffraction order, ∆*λ* is the wavelength range of the broadband spectrum, and *P*(*λ*_0_) is the power of the diffractive structure at the design wavelength *λ*_0_.

These advanced configurations help reduce LCA at the near focus in typical bifocal IOLs and at both intermediate and near foci in typical trifocal lenses. However, the distance focus (diffraction order *m* = 0) remains unaffected by the diffractive structure and thus retains the eye’s inherent LCA.

To address this limitation, novel IOL designs have emerged that incorporate taller diffractive steps, inducing a greater phase shift in the incident wavefront. In doing so, the primary distance focus is generated via the +1st diffractive order, rather than the 0th, effectively introducing a reversed chromatic dispersion at the distance focus. This design strategy, used in IOLs such as Tecnis Symfony, FineVision trifocal, and Johnson & Johnson Synergy, leverages the reversed LCA inherent in positive diffraction orders to partially offset the chromatic aberration of both the cornea and the refractive carrier lens—even at the distance focus [69,70,71]. However, shifting the distance focus to a higher diffraction order can sometimes lead to overcompensation of the eye’s natural LCA. This may introduce increased LCA at the near focus, potentially degrading image quality for close-up tasks.

As a useful benchmark, a diffractive IOL with ~3.4 D of diffractive power approximately neutralizes the LCA of a typical 60 D emmetropic eye—but only for that particular focus. This implies that a +3 to +4 D add power in a diffractive IOL can effectively correct chromatic aberration for near vision, although energy distribution across wavelengths still varies [72].

While compensating for LCA at the distance focus can enhance contrast sensitivity and distance image clarity, these benefits can be mitigated by higher-order aberrations, TCA [73,74], or increased light scatter. Additionally, taller diffractive structures introduce greater manufacturing complexity and are more prone to straylight and subjective glare [75], making fabrication precision and surface quality critical in such designs.

**Figure 16:**
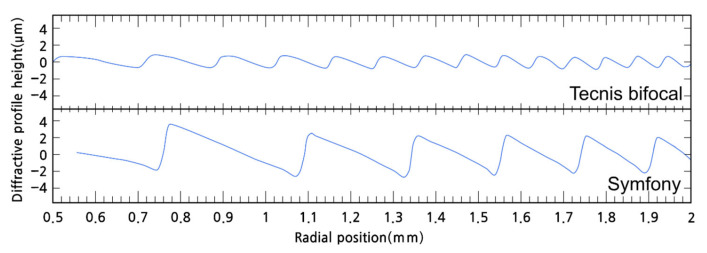
Comparison of the kinoform shapes of the Tecnis bifocal (ZMB00) intraocular lens (IOL) and the Tecnis Symfony IOL. The Symfony IOL has taller and wider kinoform structures than the Tecnis bifocal IOL. A wider kinoform results in lower addition power, while a taller kinoform leads to greater distribution of light to the 1st and 2nd orders [76].

For example, the comparison between the Tecnis bifocal (ZMB00; Johnson & Johnson) and Tecnis Symfony (Johnson & Johnson) IOL is an example. As shown in Figure 16, compared to the Tecnis bifocal, the Tecnis Symfony has nearly twice the spacing and triple the height of the kinoform [76]. Symfony has a total of 9 diffractive rings, with the central 3 rings having a height of 6.2 µm and the remaining 6 rings having a height of 5.6 µm. The 3 rings are distributed up to a diameter of 2.75 mm from the center, and all 9 rings are located within a diameter of 4.76 mm [69]. This difference in structure affects the function of the IOLs. Because the spacing of the Symfony’s kinoform is twice that of the Tecnis bifocal, its add power is half that of the Tecnis bifocal, and around 1.5D. However, the height of the kinoform is nearly triple, so unlike the Tecnis bifocal, which distributes light between the 0 order and 1st order, the Symfony primarily distributes light between the 1st and 2nd orders. This means that the height of the kinoform aims for a phase delay of one and a half wavelengths. As a result, most of the light is distributed between the 1st order (far) and the 2nd order (intermediate), thus the Symfony is classified as a diffractive EDOF design.

As shown in Table 1, for the Symfony IOL, the central 2.75 mm area has a kinoform height of 6.2 µm, causing an optical pathway difference (OPD) delay of 1.5 wavelengths, while the kinoforms in the peripheral area (outside the central 2.75 mm) cause a delay of about 1.37 wavelengths. [76] Consequently, the central area evenly distributes light between the 1st and 2nd orders, while the peripheral area distributes approximately 75% to the 1st order and 25% to the 2nd order. This design aims to direct more light to the 2nd order in a constricted pupil state rather than a dilated state. Also, it should not be overlooked that the central 1.57 mm area is designed to focus on distant objects, similar to a monofocal IOL.

However, all these calculations are based on 550 nm green light, and the situation is different for the polychromatic light we encounter in daily life. Due to the inherent spherical power of IOLs, blue light is more appropriately distributed for near vision compared to green light, while red light is more suited for distance vision.

For blue light with a wavelength of approximately 400 nm, the light distribution effect of the diffractive lens is relatively reduced. This is because the width and height of Symfony’s kinoform are designed to be suitable for 550 nm green light, making them relatively large for 400 nm blue light. Conversely, for 700 nm red light, the kinoform width and height are relatively smaller compared to 550 nm green light. As a result, blue light is more clearly distributed toward near vision, while red light is more distributed toward far-off vision. However, as previously mentioned, diffracted light bends more with longer wavelengths, meaning red light also has a tendency to shift toward near vision compared to blue light. Consequently, the overall light distribution does not change dramatically.

**Figure 17:**
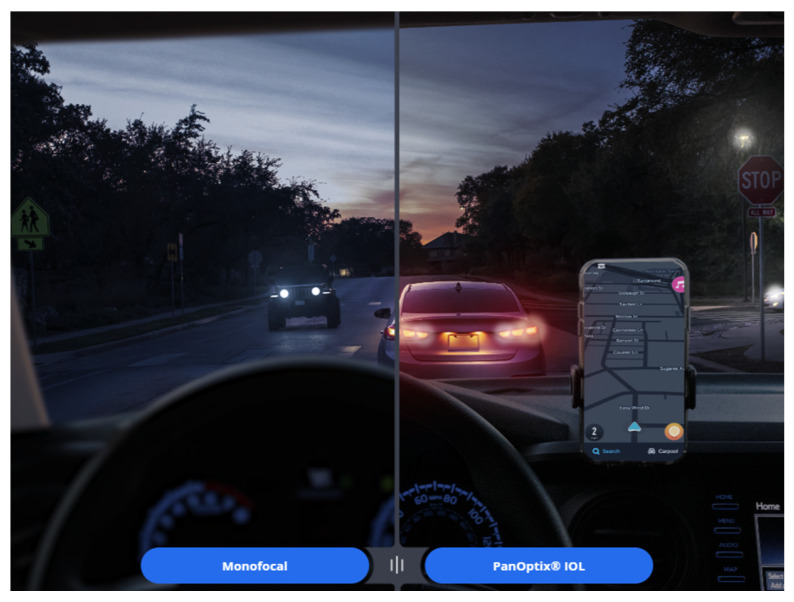
A simulator provided on the Alcon website illustrates that, compared to monofocal intraocular lenses, the PanOptix lens offers clearer vision at intermediate and near distances, but also reveals the presence of halos. In addition, distant objects appear slightly reddish.

Figure 17 illustrates a night vision simulator provided by the MyAlcon website [77], showing the visual experience after inserting the PanOptix IOL [77]. An interesting observation is that, unlike a monofocal IOL, the PanOptix lens allows focus at various distances, yet distant cars appear more prominently in red. The reason for this phenomenon, as previously explained, is that due to the kinoform height of the PanOptix lens, red light is primarily distributed toward distance vision, while blue light tends to be distributed toward near vision. Although red light undergoes more diffraction, slightly reducing its far-focus distribution effect, this phenomenon is commonly observed in most diffractive IOLs. As a result, sensitive patients may perceive distant objects appearing slightly redder and near objects appearing bluer when using diffractive IOLs [78].

## 8. The Importance of Cornea Optical Characteristics

When selecting the most suitable IOL for a patient, it is crucial not to overlook the optical characteristics of the cornea. Light must pass through the cornea and undergo certain optical modifications (refraction by the corneal anterior and posterior surfaces) before reaching the IOL. Current IOLs are designed with the goal of achieving optimal optical outcomes using light with a highly uniform wavefront pattern. It is well known, the cornea exhibits not only astigmatism, which is a low-order aberration, but also various higher-order aberrations, including spherical aberrations. These higher-order aberrations differ for each individual cornea. In clinical practice, topography devices sometimes quantify these aberrations as irregular astigmatism, and some devices separately calculate and provide corneal spherical aberration values. However, the cornea, especially in elderly patients, can exhibit significant irregular astigmatism [79,80,81]. These aberrations, often referred to as higher-order aberrations, complicate the wavefront of light passing through the cornea. When light with such complex wavefronts passes through a multifocal IOL, the resulting optical modifications can become unpredictable. The most undesirable outcome is that the higher-order aberrations induced by the cornea become even more complicated due to the diffractive or refractive nature of the multifocal IOLs. For example, if the IOL being used is an EDOF IOL with negative spherical aberration applied, and a small negative spherical aberration zone is confined to the central part of the IOL, the postoperative EDOF function may decrease if the patient’s cornea itself has a very large positive spherical aberration [82]. Conversely, if the patient’s cornea has a small positive spherical aberration or even a negative spherical aberration, the EDOF function can be enhanced [83].

## 9. Pros and Cons for Multifocal or EDOF IOLs

In a systematic review (enrolled 2230 people with data available on 2061 people (3194 eyes) published in 2016, the distance visual acuity after multifocal IOL implantation was not different from monofocal IOL implantation [84]. Spectacle independence was higher for multifocal IOL-implanted eyes, as the relative ratio (RR) for an unaided near-visual acuity worse than 0.5/0.3 was 0.20, with a 95% confidence interval (CI) from 0.07 to 0.58. However, people receiving multifocal lenses were more likely to report problems with glare (RR 1.41, 95% CI 1.14 to 1.73) compared with people receiving monovision [84]. Recent advancements in premium IOLs have been focused on minimizing dysphotopsia while providing patients with satisfactory near vision. Although patients prioritize distance vision for activities like driving, near and intermediate vision—essential for viewing displays such as monitors, smartphones, and tablets—are equally important. Selecting the appropriate IOL requires thorough consultation with the patient to accurately identify their needs. However, it is crucial to make patients aware during consultations that current IOL technology cannot fully meet all their needs. As previously discussed, due to the optical characteristics of IOLs, there are significant limitations with both EDOF and multifocal IOLs until a truly accommodating IOL—comparable to the crystalline lens of individuals in their twenties—becomes commercially available.

## 10. Conclusions

IOLs are a groundbreaking technology that significantly enhances patients’ quality of life following cataract surgery. Over the past two decades, IOLs have evolved from spherical to aspherical designs and now incorporate multifocal functionality, allowing patients to see objects at various distances. However, this advancement has come with compromises, as the exceptionally sharp image quality provided by monofocal IOLs has been partially sacrificed. Even today, IOLs continue to develop, driven by an increasing understanding of various optical phenomena. Emerging technologies—accommodating IOLs, customizable optics, and wavefront-guided lens selection—offer complementary pathways to enhance postoperative visual quality. Accommodating IOLs may restore dynamic focus via residual ciliary function, reducing reliance on multifocal designs. Customizable optics may correct patient-specific higher-order aberrations, and wavefront-guided selection using individualized aberrometry can minimize residual errors and improve contrast and night vision.

For ophthalmologists offering diverse IOL options to patients, a fundamental understanding of IOL optics is essential. This knowledge serves as a foundation for selecting the most suitable IOL for each patient and addressing any discomfort they may experience after implantation.

## Figures and Tables

**Table 1:** Kinoform heights of Symfony IOL and resultant phase differences (optical pathway difference; OPD) [76].

Echelette/Ring	Step Height (µm)		Outer Diameter (mm)		OPD (*λ*_0_ Units)
	Design	Experimental (±0.2)	Design	Experimental (±0.1)	Design
1	6.2	NA	1.60	1.57	1.5
2	6.2	6.3	2.20	2.21	1.5
3	6.2	6.2	2.75	2.72	1.5
4	5.6	5.5	3.17	3.14	1.366
5	5.6	5.7	3.55	3.53	1.366
6	5.6	5.8	3.88	3.86	1.366
7	5.6	NA	4.20	4.19	1.366
8	5.6	NA	4.48	4.46	1.366
9	5.6	NA	4.76	4.74	1.366

## Abbreviations

The following abbreviations are used in this manuscript:

CI	Confidence interval
EDOF	Extended depth of focus
IOL	Intraocular lens
LCA	Longitudinal chromatic aberration
MTF	Modulation transfer function
OPD	Optical pathway difference
PSF	Point spread function
RR	Relative ratio
TCA	Transverse chromatic aberration
